# Identification of Candidate Genes for Green Rind Color in Watermelon

**DOI:** 10.3390/plants14010113

**Published:** 2025-01-02

**Authors:** Wei Zhou, Licong Yi, Yunqiang Wang, Hongsheng Wang, Qingke Li, Na Wu, Zhaoyi Dai

**Affiliations:** 1 Hubei Key Laboratory of Vegetable Germplasm Enhancement and Genetic Improvement, Institute of Industrial Crops, Hubei Academy of Agricultural Sciences, Wuhan 430064, China; weizhou426@163.com (W.Z.); ylc881128@163.com (L.Y.); wangyunqiang0909@sina.com (Y.W.); eshukj@163.com (H.W.); liqingke1999@163.com (Q.L.); 15271222001@163.com (N.W.); 2 Key Laboratory of Ecological Cultivation on Alpine Vegetables (Coconstruction by Ministry and Province), Ministry of Agriculture and Rural Affairs, Wuhan 430063, China; 3 College of Horticulture and Gardening, Yangtze University, Jingzhou 434025, China

**Keywords:** watermelon (*Citrullus lanatus* L.), fine mapping, rind color, *ClAPRR2*

## Abstract

The color of the rind is one of the most crucial agronomic characteristics of watermelon (*Citrullus lanatus* L.). Its genetic analysis was conducted to provide the identification of genes regulating rind color and improving the quality of watermelon appearance. In this study, a mapping population of 505 F_2_ plants, derived from a cross between green (CG058) and light-green (CG265) rinds, along with a high-density genetic linkage (average 0.9 cM distance between bin markers), was used to map and identify possible candidate genes. The green rind trait was determined to be regulated by a single Mendelian locus and was precisely located within a 110 kb genomic site on chromosome nine (Chr 9). In the respective region, two potential genes, *Cla97C09G175170* and *Cla97C09G175180*, were substantially downregulated in the light-green rind in comparison to the green rind. Previous studies revealed that *Cla97C09G175170*, encoding a two-component response regulator-like protein (APRR2), is possibly involved in the green rind trait in watermelon. Virus-induced gene silencing (VIGS) assay confirmed that *ClAPRR2* is a key gene responsible for green rind color. Moreover, qRT-PCR analysis revealed that the transcription levels of multiple key genes in the chlorophyll (Chl) biosynthesis pathway were downregulated in the light-green rind relative to the green rind. The current findings have the potential to clarify the regulatory mechanisms that underlie the color of the watermelon rind. These data would provide valuable insights for the targeted molecular design and development of watermelon rinds.

## 1. Introduction

Globally, watermelon (*Citrullus lanatus* L., 2n = 2x = 22) is an essential horticultural product that is cultivated extensively. It has been designated as a model crop for the study of fruit characteristics as a result of its vast diversity in fruit-related traits, including fruit size and shape, rind thickness and color, flesh flavor and texture, and seed size and color [1]. Among these characteristics, rind color is a crucial factor that directly affects consumer choice and market value, making it the focus of breeders [2].

The color of watermelon rinds varies from yellow to light green, dark green, and gray [3]. Green tissue coloration in plants is primarily due to chlorophyll (Chl), which captures light energy for photosynthesis [4]. The chlorophyll metabolism pathway in plants has been widely studied and comprises four distinct pathways [5]. The reactions described in the first phase are catalyzed by the enzyme in multiple stages, which begin with the conversion of 5-aminolevulinic acid (ALA) to protoporphyrin IX. In the second phase, the “Mg branch” is defined as the addition of Mg^2+^ into protoporphyrin IX for Chl *a* formation [6]. This phase synthesizes Chl *a*. The third phase comprises the “Chl cycle”, which promotes the interaction of Chl *a* and *b* [7]. In the final phase, the pheophorbide an oxygenase (PAO)/phyllobilin pathway is the mechanism by which Chl *a* is degraded to produce colorless, nonfluorescent Chl catabolites (NCCs) [8].

Several studies have investigated the genetic pattern and regulatory mechanism of watermelon rind color. The depth and background of the rind color have been shown to be determined by the D and Dgo gene loci [9]. In 1956, the *go* gene was reported for the first time. This gene has a single recessive pattern of inheritance for yellow rind. Along with the go gene, a 59.8 kb QTL locus on Chr 4 was mapped to the second yellow rind gene using BSA-seq and GWAS analyses [10]. Another *yellow rind* (*Clyr*) gene, responsible for the yellow rind trait, was precisely located within a 91.42 kb region on Chr 4. However, the transcription level of the candidate genes in this region remained unchanged between the parents [11]. A solid dark-green rind color has been reported to be qualitatively inherited. The light-green color of the rind was only observed when the g-1 and g-2 genes were in homozygous recessive form [12].

Recently published high-quality watermelon reference genomes have enabled significant advancements in the mapping of watermelon rind color genes [13,14]. Moreover, the *ClCG08G017810* gene on Chr 8 has been recognized as a potential gene responsible for the color of the outer layer of watermelon [3]. Both *Cla97C09G175170* and *Cla97C09G175150* have been proposed as potential candidates for stripe and interstripe color [15]. The *Cla97C06G125710* gene on Chr 6, which encodes a chlorophyll *a*-*b* binding (CAB) protein, has been reported to be responsible for the different shades of green rind color in watermelon [16]. Oren demonstrated that the two-component response regulator-like protein (APRR2) transcription factor is crucial for the variation in fruit coloration. Its allele resulted in a qualitative difference between green and light-green rinds in both plants [17].

Watermelon rinds have been observed to have different combinations of green colors. Several studies have examined the genes associated with the color of the rind, whether light green or green. Therefore, the current study was also designed to evaluate the inheritance of watermelon rind color in six generations of crosses derived from ‘CG058’ (green) and ‘CG265’ (light green). The genetic basis and the preliminary map of the F_2_ population for the green rind locus (6.9 Mb region) were determined via SNP-panel sequencing. Molecular markers were designed to analyze the recombinants and refine the initial region for precise localization. Finally, the candidate gene in the fine-mapping region was validated using qRT-PCR and VIGS assays. These results offer new perspectives into the candidate genes that function in green rinds and may expedite marker-assisted selection.

## 2. Results

### 2.1. Genetic Inheritance of Watermelon Rind Color

**Figure 1:**
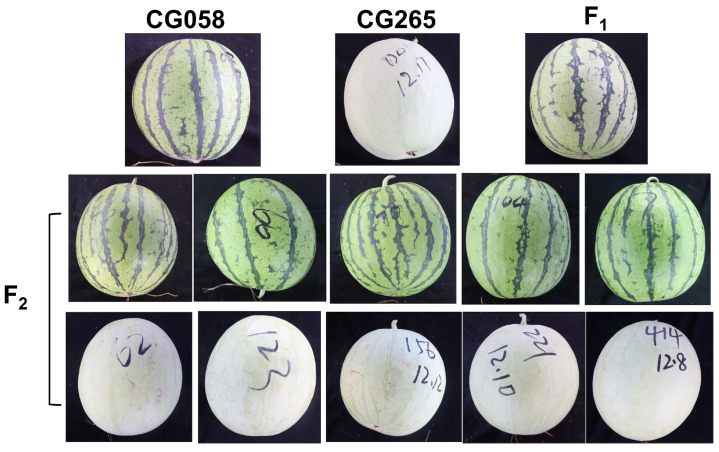
Fruit images of CG058, CG265, and their F_1_ and F_2_ progenies.

To investigate the genetic inheritance of rind color in watermelon, two parental lines were selected with distinguishable rind colors. CG058 showed a green rind color, while CG265 displayed a light-green or gray rind color. All F_1_ plants that were produced from the hybridization between these parental lines showed a green rind color without segregation, thereby confirming the dominant trait of green rind color (Figure 1). A total of 331 individuals in the F_2_ population displayed green rinds. In comparison, 101 individuals showed light-green rinds, with a 3:1 Mendelian ratio. All the individuals in the BC_1_P_1_ population revealed green rinds resembling the F_1_ plants, while 42 individuals out of the 79 plants in the BC_1_P_2_ population showed green rinds. In the BC_1_P_1_ population, the ratio of plants with green rinds to those with light-green rinds was approximately 1:1 (Table 1). The findings indicated that the green rind color was a dominant trait that was regulated by a single locus.

### 2.2. Chloroplast Observation and Chlorophyll Measurement

**Figure 2:**
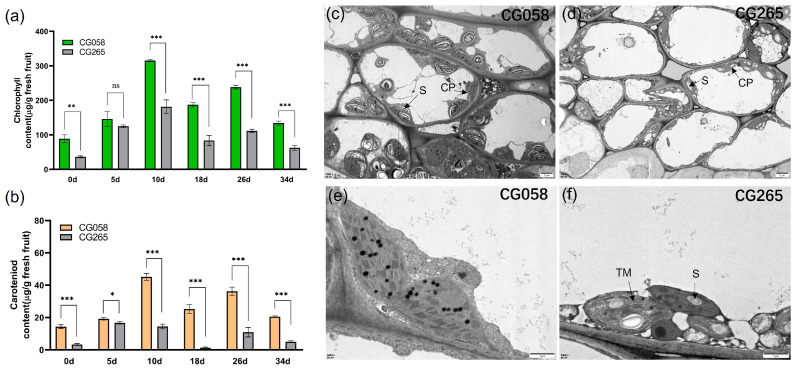
Pigment content and cytological observations of the parental line rind. (**a**) Contents of *Chl* of the parental rind. (**b**) Contents of carotenoid of the parental rind. (**c**) Transmission electron microscopy (TEM) of chloroplasts in CG058. (**d**) Transmission electron microscopy (TEM) of chloroplasts in CG265. (**e**) Chloroplast ultrastructure in CG058. (**f**) Chloroplast ultrastructure in CG265. Data are illustrated as means ± SE. * = *p* < 0.05; ** = *p* < 0.01; *** = *p* < 0.001; ns = no significance. S: starch grains; chlorophyll (Chl); CP: chloroplast; TM: thylakoid membrane.

To examine the variations in rind pigment content during fruit development, the total chlorophyll (Chl) content of the rind samples from the two parental lines was measured at various developmental phases (0, 5, 10, 16, 26, and 34 days after pollination (DAP)) (Figure 2a). The findings illustrated that the Chl content of the green rind parent (CG058) was substantially higher (1.7 to 2.2 fold) than that of the light-green rind parent (CG265) at all tested stages, except at 5 DAP, where there was no significant difference (Figure 2b). The visual observations were correlated with the Chl content measurements in the rinds of both parents. Further, the carotenoid content of the rinds of both parents demonstrated a similar trend to that observed in Chl content (Figure 2b).

The fruit rinds at 30 DAP were collected and analyzed via transmission electron microscopy (TEM) to determine the quantity and ultrastructure of chloroplasts in the rinds of both parental lines (Figure 2c–f). Based on the findings, CG058 contained more chloroplasts than the CG265 rind. Moreover, the chloroplasts in the CG058 rind were tightly packed, whereas those in the CG265 rind were irregularly arranged (Figure 2c,d). Overall, the CG058 rind contained chloroplasts that were considerably larger than those in the CG265 rind (Figure 2e,f).

### 2.3. Detection of Candidate QTLs for Rind Color via SNP Panels

**Figure 3:**
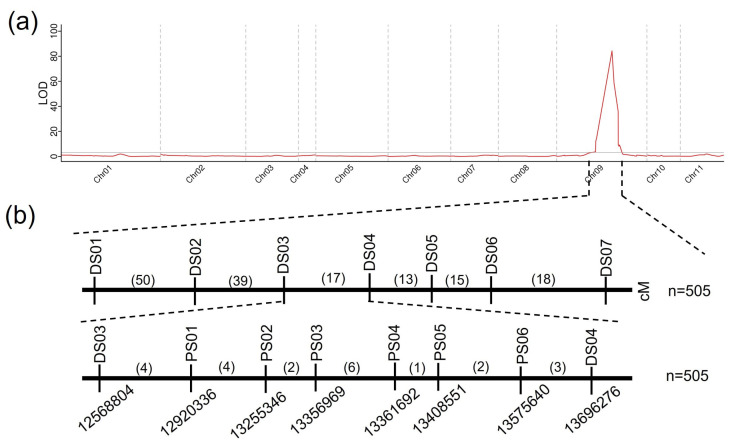

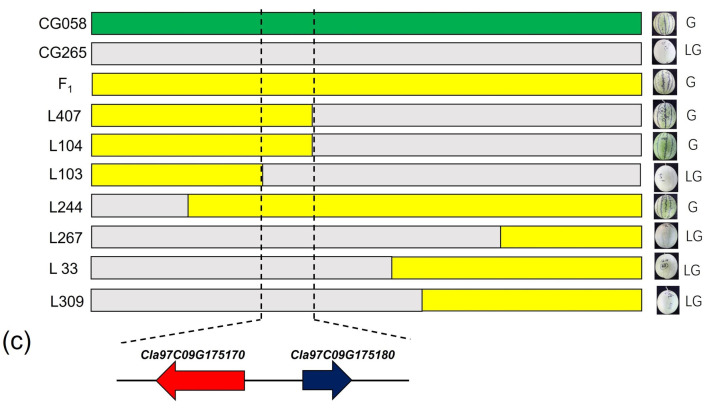
Genetic mapping of the CG058 rind gene locus. (**a**) Initial mapping of the CG058 rind gene locus. (**b**) Fine mapping of the CG058 rind gene locus. (**c**) Genes in the candidate region. Green represents the homozygous CG058 segment, gray signifies the homozygous CG265 segment, and yellow denotes the heterozygous region. Numbers above the Chr represent recombinants between adjacent markers. The number of plants used for mapping is denoted by n.

To identify the QTLs associated with rind color, an SNP panel with 213 SNP markers was used to detect the genotypic profile of the 505 F_2_ individuals. The green rind locus was mapped to a region (9.7–16.6 Mb) on Chr 9, which corresponded to the phenotype of the F_2_ individuals (Figure 3a).

Seven InDel markers were developed to further refine this region by identifying the genotypes of the 505 individuals for initial mapping via the sequence difference between the two parental lines. The green rind locus was confined to an area of 1.11 Mb between markers DS03 (12.56 Mb) and DS04 (13.69 Mb) (Figure 3b). Approximately 17 recombinants were identified among the 505 F_2_ individuals using the markers DS03 and DS04. Six new markers were developed in the region (12.56–13.69 Mb) to genotype the recombinants (Figure 3b). Finally, the gene responsible for determining the green rind color was identified in a 110-kb region between the PS02 and PS03 markers (Figure 3b).

### 2.4. Candidate Gene Sequencing and Expression

*Cla97C09G175170* and *Cla97C09G175180* are two possible genes that are present in the 110-kb region, as indicated by the annotation data of the reference genome 97103 (v2) (Figure 3c). A two-component response regulator-like protein, ClAPRR2, is encoded by *Cla97C09G175170*, whereas the function of *Cla97C09G175180* is unknown.

**Figure 4:**
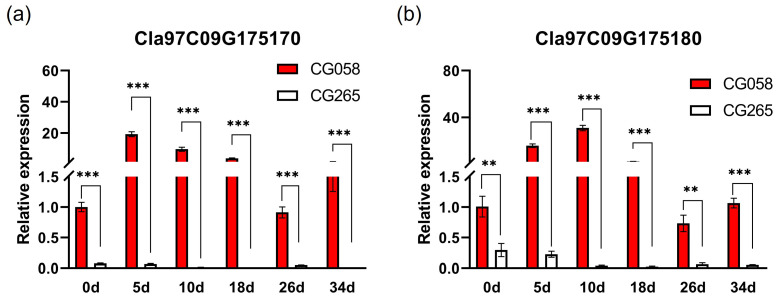
Detection of the expressions of potential genes from the parents. (**a**) Relative expression of *Cla97C09G175170*. (**b**) Relative expression of *Cla97C09G175180*. The rinds are represented by 0, 5, 10, 18, 26, and 34 d on the x-axis, indicating the days after pollination. Data are illustrated as means ± SE. ** = *p* < 0.01; *** = *p* < 0.001.

Unexpectedly, the assembled genome resequencing data did not reveal any variation between both parental lines in the selected region. After that, the transcriptional pattern of both genes was analyzed in the parental lines (Figure 4). Importantly, *Cla97C09G1750170* and *Cla97C09G175180* showed no expression in light-green rinds. The expression of both genes gradually increased during the color accumulation stage and then decreased after reaching a peak in the green rind. The expression of *Cla97C09G1750170* was the highest at 5 DAP, while *Cla97C09G1750180* had the maximum expression at 10 DAP, which was nearly 30 times higher than that in the initial stage. Therefore, this study suggests that *Cla97C09G1750170* and *Cla97C09G175180* might be responsible for the rind color trait.

### 2.5. VIGS Assay Validates ClAPRR2 as a Crucial Gene for Green Rind Color

**Figure 5:**
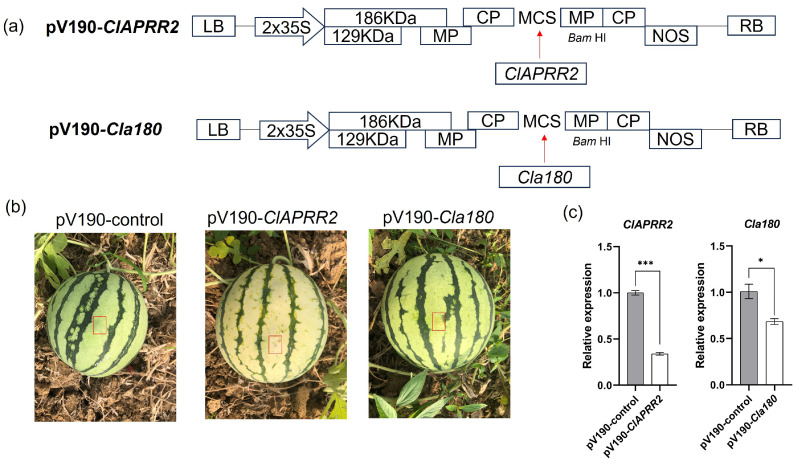
Phenotypic and gene expression of watermelon fruits infiltrated with VIGS constructs. (**a**) Maps of the pV190-*ClAPRR2* and pV190-*Cla180* vectors. (**b**) Variations in the phenotypes of fruits that were infiltrated with various VIGS constructs at 25 DAP. Infiltrated positions are indicated by red arrows. (**c**) Expression of *ClAPRR2* and *Cla180* in VIGS-treated rinds. Data are illustrated as means ± SE. * = *p* < 0.05; *** = *p* < 0.001.

Various fruits, including blueberry [18], banana [19], and loquat [20], have been extensively tested using the VIGS assay. The function of *ClAPRR2* and *Cla97C09G175180* (*Cla180*) was validated by inserting a 220-bp fragment from *ClAPRR2* and a 224-bp fragment from *Cla97C09G175180* into the *cucumber green mottle mosaic virus* vector (pV190) to produce pV190-*ClAPRR2* and pV190-*Cla180*, respectively (Figure 5a). The negative control consisted of empty pV190-infected plants (pV190-*control*). *ClAPRR2* silencing resulted in photobleaching of the fruit rind in pV190-*ClAPRR2* around the infiltrated area (Figure 5b). However, the fruit rind color of pV190-*Cla180* did not show a substantial change compared to the pV190-control. *ClAPRR2* gene expression in pV190-ClAPRR2 was remarkably downregulated in qRT-PCR analysis (Figure 5c). These findings confirmed that *ClAPRR2* is a crucial gene for the development of green rind color.

### 2.6. Downregulated Genes Associated with Chl Biosynthesis Pathway in Light-Green Rind

**Figure 6:**
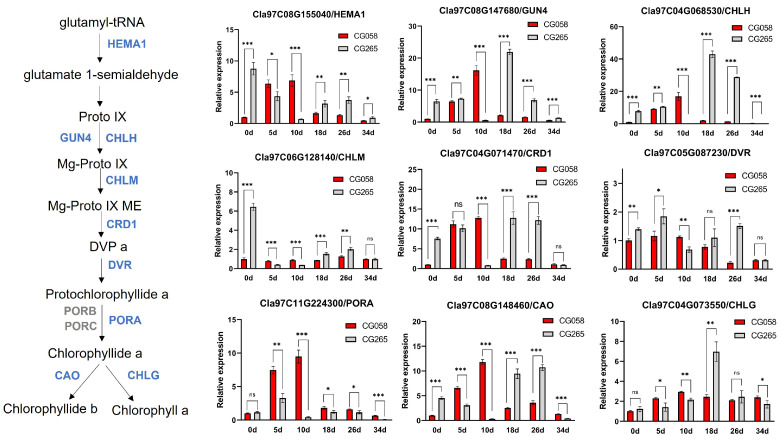
Comparative expression of the Chl synthesis pathway genes in both rind parents. The rinds are represented by 0, 5, 10, 18, 26, and 34 d on the x-axis, indicating the days after pollination. Data are illustrated as means ± SE. * = *p* < 0.05; ** = *p* < 0.01; *** = *p* < 0.001; ns = no significance.

Glutamate 1-semialdehyde is the precursor to the tetrapyrrole pathway, which deviates at Proto IX. Therefore, the relative expression of glutamyl-tRNA reductase (*HEMA1*), which catalyzes the conversion of glutamyl-tRNA to glutamate 1-semialdehyde, was determined in the rind of the two parental lines (Figure 6). The expression of *HEMA1* (*Cla97C08G155040*) in the CG058 rind was substantially higher than that in the CG265 rind at 5 DAP and 10 DAP. The branch leading to Chl accumulation, which was promoted by Mg-chelatases (GENOMES UNCOUPLED 4, *GUN4*, and Mg-chelatase subunit H, *CHLH*), was also investigated. The expression of *GUN4* (*Cla97C08G147680*) and *CHLH* (*Cla97C04G068530*) was higher only in the CG058 rind relative to the CG265 rind at 10 DAP. Mg protoporphyrin IX methyltransferase (*CHLM*), Mg-protoporphyrinogen IX monomethylester cyclase (*CRD1*), and protochlorophyllide reductase (*PORA*) are responsible for converting Mg-Proto IX to chlide *a*. The gene levels of *CHLM* (*Cla97C06G128140*) and *CRD1* (*Cla97C04G071470*) were similar to those of *HEMA1*, showing a higher expression level in the CG058 rind than in CG265 rind at 5 DAP and 10 DAP. The CG058 rind showed substantially higher levels of *PORA* (*Cla97C11G224300*) expression than the CG265 rind at all developmental phases. Chlorophyllide *a* oxygenase (*CAO*, *Cla97C08G148460*), which oxidizes chlorophyllide *a* to chlorophyllide *b*, had a similar expression pattern to that of *CHLM* and *CRD1*. Except for *PORA*, the expression of nearly all genes was higher in the CG265 rind than in the CG058 rind after 18 DAP. These results suggest that chlorophyll accumulation occurs in the early stage of green rind development.

## 3. Discussion

The physical characteristics of watermelon, including its color, shape, and stripe pattern, are crucial factors that impact the purchasing decision of the consumer. A high-quality appearance can substantially increase the market demand for watermelon. Among these, rind color is more essential. Despite several studies on the inheritance of watermelon rind color, only a few candidate genes associated with this trait have been identified [3,10,11,15,16,17]. The complexity of the genetic patterns and developmental mechanisms of rind color arises from the ambiguous terms used to characterize them and the changes in gene expression depending on the genetic backgrounds used in different studies.

This study examined the genetic pattern of rind color by crossing two watermelon germplasms, CG058 and CG265. The results showed that CG058 rinds were more dominant than CG265 rinds. In a six-generation genetic population, further genetic analysis of the rind color trait demonstrated that the CG058 rind trait in watermelon is inherited as a single dominant gene. This result is in line with the genetic profiles of horticultural crops, including cucumber (*Cucumis sativus* L.) [21], wax gourd (*Benincasa hispida*) [22], eggplant (*Solanum melongena* L.) [4], and melon (*Cucumis melo*) [17]. Measurements of Chl and carotenoid contents in both parental plants revealed that the CG265 rind parent had lower Chl and carotenoid contents than the CG058 rind parent. Moreover, TEM analysis evidenced that the number of CPs in the CG265 rind parent was lower than that in the CG058 rind parent. These results are similar to those reported by Li et al. [3].

Moreover, pigment content and formation determine the color of leaves and peels [23]. In watermelon, the Chl content is the main factor affecting the CG058 rinds [3]. Transcription factors have been reported to regulate chlorophyll and carotenoid accumulation, resulting in different rind colors [15]. Recently, the Golden2-like (*GLK2*) transcription factor and *APRR2*-like genes have been established as critical transcription mediators in regulating plastid metabolism across various species. *GLK* has been reported to regulate Chl levels in pepper (*Capsicum*) [24]. Similarly, *APRR2* has been identified as a regulator of pigment deposition in tomatoes and peppers, while its overexpression in tomatoes has been shown to increase the number of plastids and color intensity of fruits [25]. This gene has also been found to play a substantial role in determining white immature rind color (w) in cucumber [21], as well as in the coloration of muskmelons [17], watermelons [17], wax gourds [22], bottle gourds (*Lagenaria siceraria*) [26], and eggplant peels [4]. As expected, *ClAPRR2* was located in the fine-mapping region, similar to previous research. The VIGS assay in watermelon rind validated that *ClAPRR2* is critical for the green rind color. This study also investigated the transcription pattern of genes responsible for Chl biosynthesis. The CG058 rind parent showed higher expression of nearly all genes than the CG265 parent before 10 DAP, suggesting that Chl accumulation occurred at an initial phase.

It was reported that DNA methylation controls gene expression by either blocking the interaction of transcription factor(s) to DNA or recruiting suppressor proteins [27]. In Radish (*Raphanus sativus*), the hypermethylated CACTA transposon induced DNA methylation in the promoter region of *RsMYB1*, resulting in a white-fleshed phenotype [28]. In Chrysanthemum (*Chrysanthemum morifolium*), the methylation status of the *CmMYB6* promoter was highly correlated with the flower color, and this methylation was heritable [29]. In the dominant waxless cabbage (*Brassica oleracea* L. var. *capitata*) mutant, the causative gene *non-wax glossy* (*NWGL*) was located in a 99 kb region at the end of cabbage Chr C08. However, this region did not yield any DNA variations in the potential gene (*Bol018504*). Moreover, the mutant phenotype was possibly due to lower expression and modified DNA methylation [30]. The yellow rind in watermelon may also have been affected by methylation in the WT4 cultivar [11]. This study suggests that the light-green feature of CG265 may also be a consequence of methylation. However, further research is necessary to determine its crucial molecular mechanism. Collectively, this study establishes a primary research line for further assessing the regulatory mechanisms of color genes in the fruit rinds of watermelon.

## 4. Materials and Methods

### 4.1. Plant Materials

CG058 (P_1_) and CG265 (P_2_) are germplasm obtained from the Industrial Crops Institute of Hubei Academy of Agricultural Sciences, with green and light-green rind colors, respectively. The F_1_ plant population was derived by crossing CG058 and CG265. The female flower of CG058 was pollinated with the male flower of CG265, and the seeds obtained from CG058 were the F_1_ progeny. The F_2_ population originated from the self-pollination of the F_1_ population. The backcross populations BC_1_P_1_ and BC_1_P_2_ were derived by crossing F_1_ with each parent. All plants were grown in an open field at the experimental base of the Hubei Academy of Agricultural Sciences in Hainan Province (Sanya, Hainan) in November 2021. The soil type is clayey. The daily temperature was maintained between 20 °C and 30 °C and the relative humidity was between 50% and 80%. Drip irrigation tape was used for irrigation. A total of 0.5 kg of commercial organic fertilizer and 0.06 kg of potassium sulfate compound fertilizer (N:P:K ratio of 15:15:15) per square meter was applied. As the fruit matured, the green and light-green rind phenotypes were visually observed. The rind phenotype of F_2_ was determined by comparison with the rinds of CG058, CG265, and their F_1_ progeny. In the F_2_/BC_1_ population, the segregation ratios of green/light-green rinds were analyzed via Chi-square tests (c2).

### 4.2. Pigment Content and Chloroplast Observation

The same method of pigment extraction was used as described previously [25]. The fruit rinds at different stages (0, 5, 10, 18, 26, and 34 DAP) were crushed into powder in liquid nitrogen and extracted with anhydrous ethanol for 24 h in the dark. The sample was centrifuged at 6000 rpm for 10 min, and the supernatant was measured at different 665, 649, and 470 nm wavelengths via a microplate reader (Infinite 2000, Tecan). The pigment content was calculated as mentioned previously [31]. The method of CP observation was described previously [3]. The fruit rinds at 30 DAP of both parents were collected, cut into 1 to 2 mm^3^ pieces, and fixed immediately in precooled fixative (2.5% glutaraldehyde). After fixation, dehydration, infiltration, and embedding steps, the sample was sectioned into 50 to 70 nm ultra-thin sections using an ultramicrotome (Leica UC7; Leica, Wetzlar, Germany) and observed under a Tecnai G2 20 TWIN (FEI, USA) TEM.

### 4.3. Genome Resequencing and Variant Calling

The procedure of genome resequencing and variant calling has been reported previously [32]. Briefly, genomic DNA was isolated with a CTAB method [33]. A DNA library was developed using the genomic DNA of CG058 and CG265 and then sequenced on the HiSeq 4000 (Illumina, San Diego, CA, USA) platform. Adapters and low-quality reads were removed via the Fastp software (v0.23.0) [34]. The average mass number was calculated using a sliding window of 4 bp, and all subsequent bases were removed if they were less than 15. The length of clean reads must be longer than 50 bp. The clean reads were mapped to the watermelon reference genome 97103 (v2) (http://cucurbitgenomics.org/organism/21, accessed on 27 June 2019) using the MEM algorithm of the Burrows–Wheeler Aligner (BWA, v0.7.15-r1140) software. The alignment results in SAM format were obtained. Then, the SAM format file was converted to BAM format using Samtools software (version 1.3.1), and the reads in the BAM file were sorted. The final BAM file was used for variant calling. The HaplotypeCaller module in the GATK (version 3.7) software package was used to generate GVCF files for each sample, and then the GenotypeGVCFs module was used to perform mutation detection on all samples together, including SNPs and InDel [35].

### 4.4. Marker Development and Genetic Mapping

An SNP panel containing 198 SNPs and 15 InDels was developed as a preliminary map of the green rind locus. Initially, the SNP panel was used to genotype the F_2_ population, which consisted of 505 individuals resulting from the cross between CG058 and CG265. Composite interval mapping (CIM) was used for QTL analysis on the F_2_ population, as indicated by their phenotypic characteristics, using the WinQTLCart2.5 software [36]. To narrow the mapping interval (Chr09:97.8 to 166.12 Mb), seven InDel markers (DS01-DS07) were designed for each 0.4–1.8 Mb interval, as per the SNP density, and used to genotype the 505 F_2_ individuals. The recombinant plants were genotyped using six SNP markers (PS01-PS06), each with an average interval of 100 kb, to fine-map the green rind locus. The TransStart^®^ FastPfu PCR SuperMix (TransGen Biotech, Beijing, China) was used for PCR amplification under the following conditions: step 1, 98 °C for 1 min; step 2, 98 °C for 10 s, 55–60 °C for 5 s, 72 °C for 30 s, (30–35 cycles); and step 3, 72 °C for 1 min. For the CAPS markers, the PCR product was digested with appropriate restriction endonucleases and separated by 1.0% agarose gels. The PCR product was separated via 8% gel electrophoresis (PAGE) for InDel markers. The sequences of the used markers are listed in Table S1.

### 4.5. Gene Expression via qRT-PCR Analysis

Total RNA was isolated from the rind of two parental lines at different developmental stages (0, 5, 10, 18, 26, and 34 DAP) using a FastPure Universal Plant Total RNA Isolation Kit (RC411-01, Vazyme, Nanjing, China) according to the operating instructions. Further, cDNA was synthesized via the TransScript First-Strand cDNA Synthesis SuperMix (TransGen Biotech, China). qRT-PCR analysis was conducted using the PerfectStart Green qPCR SuperMix (TransGen Biotech, China) and the CFX384 system (Bio-Rad, Hercules, CA, USA).

To normalize the Ct values, the gene *ClActin* (*Cla97C02G026960*) was used as the internal control [32]. Each experiment was performed in three biological and three technical replicates. The expression was analyzed using the 2^−ΔΔCt^ method [37]. The details of genes with primer sequences are provided in Table S2.

### 4.6. VIGS Vector Construction and Agrobacterium Infiltration

To analyze the candidate genes related to rind color in watermelon, a pV190-based VIGS [38,39] assay was conducted. *Agrobacterium tumefaciens* strain GV3101 was transformed by inserting the intact coding sequence (200–300 bp) of the desired gene into the Bam HI restriction site of the pV190 vector. The strains were freshly grown on lysogeny broth (LB) medium enriched with appropriate antibiotics and cultured overnight at 28 °C. To obtain an OD_600_ of 0.8–1, the bacteria were resuspended in an inducing buffer that contained (10 mmol/L MES, 10 mmol/L MgCl_2_, and 100 μmol/L acetosyringone). A 1.0 mL needleless syringe was used to infiltrate the cells into the rind of the watermelon after being kept at 25 °C for 3 h in the dark. The empty pV vector served as a negative control. The injected watermelon was bagged for 3 days. Each inoculation was repeated in triplicate. The transcription levels of the target genes were quantified by collecting the rind until the phenotype was observed. The primers used for the construction of the VIGS vector are provided in Table S3.

## 5. Conclusions

In the current study, we mapped the green rind color gene of watermelon to chromosome 9. The candidate gene *Cla97C09G1750170* (*ClAPRR2*), which encodes a two-component response regulator-like protein, was validated to be a crucial gene for green rind color. Our study provides valuable insights into the targeted molecular design and development of watermelon rinds. Further research will be conducted into the regulatory mechanisms of *ClAPRR2* in rind color.

## Figures and Tables

**Table 1:** Rind color segregation in 6 generations of crosses (CG058 × CG265).

Generation	Total	Green	Light Green	Ratio	*χ*^2^ Value	*p* Value
P_1_ (CG058)	10	10	0			
P_2_ (CG265)	10	0	10			
F_1_ (P1 × P_2_)	15	15	0			
F_1_ (P2 × P_1_)	15	15	0			
F_2_	505	383	122	3:1	0.094	0.666
BC_1_P_1_	37	37	0			
BC_1_P_2_	57	25	32	1:1	0.431	0.354

## Data Availability

The resequencing data of CG058 and CG265 are available on the NCBI website (https://www.ncbi.nlm.nih.gov/biosample/, accessed on 17 October 2021) under the accession numbers SAMN22357444 and SAMN22357441, respectively.

## Supplementary Materials

The following supporting information can be downloaded at: https://www.mdpi.com/article/10.3390/plants14010113/s1, Table S1: Molecular biomarkers for fine mapping; Table S2: Primer information used for qRT–PCR analysis; Table S3: Primers used for constructing VIGS vectors.

## Abbreviations

The following abbreviations are used in this manuscript:

bp	Base pair
cM	Centimorgan
SNP	Single nucleotide polymorphism
CAPS	Cleaved amplified polymorphic sequence
InDel	Insertion-deletion
LOD	Log-likelihood
DAP	Days after pollination
Chl	Chlorophyll
CP	Chloroplast
VIGS	Virus-induced gene silencing
